# Neutralizing antibodies against porcine epidemic diarrhea virus block virus attachment and internalization

**DOI:** 10.1186/s12985-018-1042-3

**Published:** 2018-08-30

**Authors:** Lang Gong, Ying Lin, Jianru Qin, Qianniu Li, Chunyi Xue, Yongchang Cao

**Affiliations:** State Key Laboratory of Biocontrol, School of Life Sciences, Sun Yat-sen University, Guangzhou, China

**Keywords:** Porcine epidemic diarrhea virus, S protein, Neutralizing antibody, Attachment

## Abstract

**Background:**

Porcine epidemic diarrhea virus (PEDV) is emerging as a pathogenic coronavirus that causes a huge economic burden to the swine industry. Interaction of the viral spike (S) surface glycoprotein with the host cell receptor is recognized as the first step of infection and is the main determinant of virus tropism. The mechanisms by which neutralizing antibodies inhibit PEDV have not been defined. Isolating PEDV neutralizing antibodies are crucial to identifying the receptor-binding domains of the viral spike and elucidating the mechanism of protection against PEDV infection.

**Methods:**

B cell hybridoma technique was used to generate hybridoma cells that secrete specific antibodies. *E.coli* prokaryotic expression system and Bac-to-Bac expression system were used to identify the target protein of each monoclonal antibody. qPCR was performed to analyze PEDV binding to Vero E6 cells with neutralizing antibody.

**Results:**

We identified 10 monoclonal antibodies using hybridoma technology. Remarkably, 4 mAbs (designed 2G8, 2B11, 3D9, 1E3) neutralized virus infection potently, of which 2B11 and 1E3 targeted the conformational epitope of the PEDV S protein. qPCR results showed that both 2B11 and 2G8 blocked virus entry into Vero cells.

**Conclusion:**

The data suggested that PEDV neutralizing antibody inhibited virus infection by binding to infectious virions, which could work as a tool to find the receptor-binding domains.

**Electronic supplementary material:**

The online version of this article (10.1186/s12985-018-1042-3) contains supplementary material, which is available to authorized users.

## Background

Porcine epidemic diarrhea virus (PEDV), which belongs to the Alphacoronavirus genus of the Coronaviridae family, is an etiological agent of porcine epidemic diarrhea (PED) and causes an enteric disease that affects all ages of swine [1, 2]. The clinical presentations and complications of infection are characterized by acute vomiting, dehydration, watery diarrhea, and high mortality in sucking piglets [3] and are indistinguishable from those of infection by either transmissible gastroenteritis virus (TGEV) or porcine enteric alphacoronavirus (PEAV) [4, 5].

First detected in the UK in 1971, PEDV resulted in mass epidemics within Europe in the 1970s and 1980s [6]. Before 2013, PED was prevalent in Asia and Europe [1]. After spring 2013, however, PED outbreaks reached North America, which was due to variant PEDV strains that researchers revealed might derive from Chinese variants [7, 8]. In spite of widespread immunization with the currently marketed vaccine, PED still persists in swine raising countries and resulted in devastating damage to the pork producers [9].

PEDV is an enveloped single-strand RNA coronavirus with a 28 kb genome, which includes 4 open reading frames encoding spike (S), envelope (E), membrane (M), nucleocapsid (N), as well as 3 open reading frames encoding replicase 1a, 1b and ORF3 [10]. As known for other coronaviruses, the three PEDV S glycoproteins form a club-shaped functional S trimer, which is localized on the surface of the virion and mediates essential biological functions, such as membrane fusion and receptor binding. The S protein is also responsible for the induction of nAbs and protective immunity, making it an appropriate candidate for developing an effective vaccine and diagnostic reagents [1, 11, 12]. In addition, variation in the S gene leads to antigenic diversity, and thus the S protein is useful in evaluating genetic diversity [13].

Little has been known about the components of the immune system that are effective in the protection of a pig against PEDV infection. The quantity of nAbs generated by vaccination correlates with the degree of protection against many diseases [14]. Considering the significance of nAbs in providing protection, understanding the mechanism of neutralization is necessary for development of a vaccine that elicits strong nAbs. The fragment antigen-binding (Fab) domain binds to specific pathogen targets, which prevents microbial interactions with host cell receptors and thus blocks infection [15, 16]. The protection of nAbs results from blocking interaction of free virus particles with target cell receptors. Additionally, for other nAbs, infection can be blocked through inhibiting critical intracellular processes, for example rotavirus transcription [17], nuclear translocation of human papilloma virus DNA [18], adenoviral uncoating [19], or measles virus assembly [20]. Several studies demonstrated that spike mAb can neutralize PEDV [21–26]. These studies mainly focused on locating the neutralizing domains of PEDV S protein, however, the mechanisms by which spike nAb neutralize the virus have not been defined completely. To fill this knowledge gap, we generated four mAbs that exhibited potent neutralizing activity against PEDV in vitro. Notably, 2B11 and 2G8 were found to block PEDV entry into Vero cell.

## Methods

### Cells, viruses and reagents

The Vero E6 cell line was cultured and maintained at 37 °C in DMEM containing 10% FBS and antibiotics (100 U/mL of penicillin and 100 μg/mL of streptomycin) (Solarbio, Beijing, China). Sf9 insect cell line was maintained as suspension in serum-free SF900II medium at 27 °C in spinner flasks at a speed of 90 to 100 rpm. PEDV-GDS01 (KM089829.1) and PEDV-GDS03 (AB857235.1) were propagated in Vero cells with 10 μg/mL trypsin. PEDV strain used in this study indicated GDS01 strain unless otherwise noted.

### Preparation and purification of PEDV virus antigen

PEDV was propagated and purified as in previously described [27]. Briefly, Vero cells were washed twice with phosphate buffered saline (PBS) to remove residual DMEM, followed by 1 h incubation with PEDV at 37 °C and wash with PBS. Next, the cells were infected by the virus via addition of DMEM containing 10 μg/mL of trypsin. The cells were harvested at 36 h after infection, when all cells showed characteristic cytopathogenic effect. Three cycles of freeze-thaws were done to release the intracellular virus particles, and a 30-min centrifugation at 10000 g was performed to pellet cellular debris. After clarification, the supernatant was enriched 100 times by the ultracentrifugation at 30000 g and then purified by sucrose density gradient centrifugation using sucrose solutions at: 20%(*w*/w), 40% (w/w) and 60% (w/w), respectively. The purified products were analyzed by SDS-PAGE and western blot.

### Development and purification of PEDV monoclonal antibodies

Standard procedures were used to generate hybridoma cells that secrete PEDV-specific antibodies [28] with some modifications. Briefly, female BALB/c mice (6 weeks) were immunized with the purified PEDV inactivated by β-propiolactone in complete Freund’s adjuvant (Sigma, St. Louis, MO, USA). The mouse was immunized with the purified PEDV containing of 10 μg spike protein determined through SDS-PAGE and gray scanning. Two booster immunizations were administered at 2-week intervals with PEDV in incomplete Freund’s adjuvant (Sigma, St. Louis, MO, USA). Next, the mice were sacrificed following a 3-day booster inoculation by intraperitoneal injection. PEG1450 [50% (*v*/v)] (Sigma, St. Louis, MO, USA) was used for fusion of spleen cells from immunized mice with sp2/0 myeloma cells, and hybridoma cells was cultured in 96-well plates at 37 °C in HAT (Sigma, St. Louis, MO, USA) screening culture medium. Positive hybridoma clones were picked by indirect immunofluorescence assay (IFA), followed by cloning via limiting dilution for at least three rounds. Polyclonal antibodies against PEDV were taken as positive control and normal mouse serum was taken as a negative control. Mouse Monoclonal Antibody Isotyping Reagents (Sigma, St. Louis, MO, USA) were used for the identification of the subtype of mAbs secreted by the final hybridoma clones. Ascites fluid was collected from primed BALB/c mice with paraffin oil and purified using Protein G Sepharose™ 4 Fast Flow (GE Healthcare, Pittsburgh, USA) according to the manufacturer’s instructions. Purified mAb was quantified by BCA kit (Thermo fisher, USA).

### Indirect immunofluorescence assay (IFA)

The supernatant of hybridoma cell cultures was screened for the presence of PEDV-specific mAbs by IFA. For this, primary Vero cells were grown to 100% confluency in 96 well plates and infected with GDS01 for 36 h at a multiplicity of infection (MOI) of 0.1. After fixation with 4% paraformaldehyde, the monolayers were permeabilized with 0.5% triton X-100, followed by a 1 h incubation with the supernatants of the hybridoma cell at 37 °C. Then, unbound antibodies were removed by washing with PBS and specific mAbs were detected with Cy3-conjugated Affinipure Goat Anti-Mouse IgG (H + L) (Proteintech, Rosemont, USA).

### Identification of the target protein of monoclonal antibody

In order to determine the conformational epitopes bound by the mAbs, the main structural proteins of PEDV, including SP (the S1 and partly S2 gene fragment, 1-954aa), N, M, and ORF3, were expressed by Bac-to-Bac expression system (Invitrogen Carlsbad, CA) following the manufacturer’s instructions. The sf9 cells were infected with recombinant baculovirus (MOI = 5), followed by fixation, permeabilization and incubation with supernatants of the hybridoma cells 3 days later, reactivity of the mAbs with recombinant proteins was measure with an IFA.

In order to determine the linear epitopes bound by the mAbs, truncated SP and full length of N genes were also cloned into pET-32a, the details of 7 truncated SP proteins refer to previous study [29]. The recombinant DNA was then used to transform BL21 cells for the following protein expression. Referring to the manufacturer’s instructions, Ni-Chelating Sepharose Fast Flow (GE, USA) was used for the purification of proteins by affinity chromatography. Purified protein was quantified by BCA kit (Thermo fisher, USA). The reaction of mAbs with truncated SP and N protein was evaluated by ELISA. Briefly, 96-well plates (Griener, Germany) were coated with the purified protein (100 ng) at 4 °C overnight, and then blocked with 5% milk for 1 h. After washing three times with PBS, 100 μL supernatant was added and the sample was incubated at 37 °C for 1 h. Subsequently, the plates were washed with PBS and incubated with HRP-conjugated goat anti-mouse IgG (Proteintech, USA) at 37 °C for 1 h. The absorbance was measured at 450 nm. All samples were repeated three times and the sample was considered positive when the relation OD sample/OD negative control was higher than 2.1.

### Neutralization assay

To determine whether an antibody had neutralization activity, we conducted the virus neutralization test as previously described [30], with modifications. Briefly, after a 30 min inactivation at 56 °C, the test mAbs (diluted to 80 μg/mL) were filtered using a 0.22-μm membrane, followed by two-fold serial dilution. The PEDV GDS01 strain (titer: 100pfu/0.5 mL) was mixed with diluted mAb of an equal volume. The mixture was then added with trypsin (10 μg/mL), followed by 1 h incubation at 37 °C. Next, Vero cell monolayers in 6-well plates were cultured with the mixture (1 mL). After a 1 h adsorption at 37 °C, the inocula were discarded. Next, the plates were washed three times with PBS. DMEM with trypsin (10 μg/mL) was added to each well and plates were incubated at 37 °C for 48 h. The plaque was colored by the neutral red (0.03%). The Serum-neutralization (SN) titer was determined according to the highest mAb dilution, which led to inhibition of formation of viral plaque completely. Neutralization (%) were calculated using the following formula: 1- sample plaque counts/negative control counts.

To determine whether 2G8 and 2B11 could neutralize the infection of GDS03, an IFA neutralization assay was performed. 80 μg of mAb was incubated with an equal volume of 500 TCID_50_/mL PEDV for 1 h at 37 °C. Then, the sample-virus mixture was transferred to duplicate wells of a 6-well plate containing confluent Vero E6 cells. The plates were incubated at 37 °C for 1 h and then washed gently with PBS to remove unbound viruses, following with 36 h incubation at 37 °C in a 5% CO_2_ atmosphere. The PEDV-infected cells were fixed with 4% paraformaldehyde and analysed by IFA. 9G11 was used as a detective antibody.

### Analysis of PEDV binding to Vero E6 cells with nAb

Virus infection in the presence or absence of antibody was quantified as previously described, with slight modifications [22]. Diluted antibodies (2B11/100 μg/mL and 2G8/200μg/mL) were mixed with PEDV (1000pfu/mL) of an equal volume, followed by a 1 h incubation at 37 °C. Next, the mixture of antibody-virus was added to triplicate wells of confluent Vero E6 cell monolayers for a 1 h infectious adsorption at 37 °C. To analyze PEDV and nAb binding at two different time points, PEDV was incubated with Vero E6 cells for one hour at 4 °C, followed by addition of antibodies and a 1 h incubation at 37 °C. Trypsin was added throughout the experiment. The cells were washed twice with PBS, and collected for measuring cell-associated PEDV via viral RNA RT-qPCR. Briefly, the cells from each well were obtained after centrifugation at 10,000 rpm for 10 min. RNA was extracted from cells using a TRIzol reagent (Invitrogen, USA) and cDNA was synthesized with 2 μg of RNA using RT-PCR kit (TaKaRa, China). The specific primers (sense: 5’-GAATTCCCAAGGGCGAAAAT-3′; antisense: 5’-TTTTCGACAAATTCCGCATCT-3′) and probes (5’-FAM-CGTAGCAGGCTTGCTTCGGACCCA-BHQ-3′) were designed to amplify and detect the n gene of PEDV. Real-time PCR assays were carried out in 20 μL reaction mixture containing 10 μL of Thunderbird Probe qPCR Mix, 1 μL of cDNA template, 0.04 μL of 50× Rox reference dye, 0.2 μM of probe, and 0.3 μL of primers. The PCR amplification was performed with an Applied Biosystem 7500 Fast instrument (Life Technologies, USA) under the following conditions: 95 °C for 20 s for initial denaturation followed by 40 cycles of 95 °C for 3 s and 60 °C for 30 s. Ten-fold serial dilutions of standard plasmid pET-19 T-N, ranging from 10^7^ to 10^2^ copies/μL, were tested in five replicates with real-time RT-PCR to generate the standard curve.

### Competitive binding ELISA

Ninety six-well plates (Griener, Frickenhausen, Germany) were firstly coated by PEDV at a density of 10^6^ virions/well at 4 °C overnight, followed by a 1 h block with 4% BSA. After washed with PBS for three times and added with 100 μL diluted mAb (1:100), the sample was incubated for 1 h at 37 °C. Next, the plates were rinsed with PBS and cultured for 1 h with mAb 2G8 which labelled with horseradish peroxidase by EZ-Link Maleimide Activated Horseradish Peroxidase Kit (Thermo scientific, USA). The reacting results were visualized using tramethylbenzidine (TMB), stopped by HCL. All samples were repeated three times. The absorbance was determined using a microplate reader (Bio-Tek) at 450 nm.

## Results

### Generation of monoclonal antibodies

**Fig. 1:**
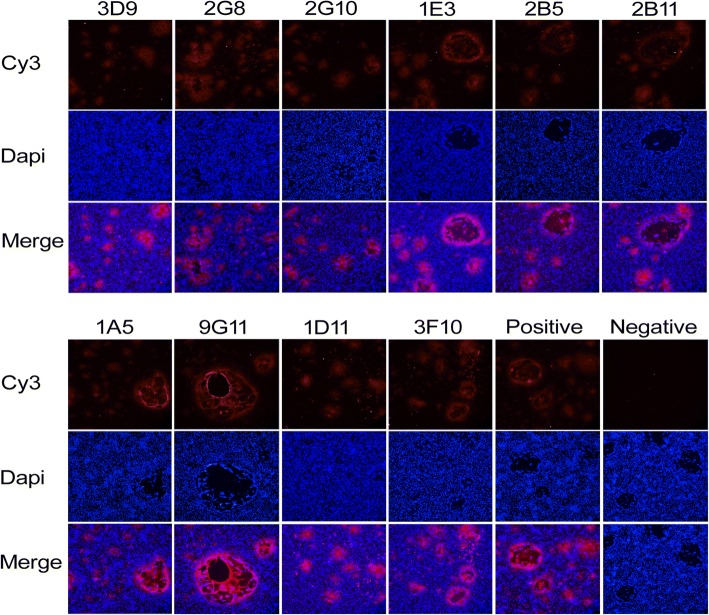
Reactivity of mAb with PEDV-infected cells, as detected by IFA. The microscopic channel (Cy3 and Dapi) used for photography is shown on the left and the different antibodies used for the assay are indicated at the top. A PEDV polyclonal antibody and a negative serum from normal mouse were used as the primary antibody, set as positive and negative control, respectively. The experiment was repeated more than three times, and representative images are shown

PEDV concentration was enriched 100 fold and purified by sucrose density gradient centrifugation. The purified products were analyzed by SDS-PAGE and western blot, and the results showed that the main structural proteins were reserved after ultracentrifugation (see Additional file 1: Figure S1). BALB/c mice were immunized with purified PEDV and ten mAbs were prepared via lymphocyte hybridoma technique, which could react well with the PEDV-infected Vero cells by IFA. As shown in Fig. 1, the syncytium formed due to PEDV infection of cells were not detected using negative mouse serum, but were specifically stained with the mouse polyclonal antibodies and 10 mAbs. All mAbs were determined to be IgG1/kappa isotype, with the exception of 1A5 (IgG3/kappa isotype) (data not show).

### Identification of the target protein recognized by mAbs

**Fig. 2:**
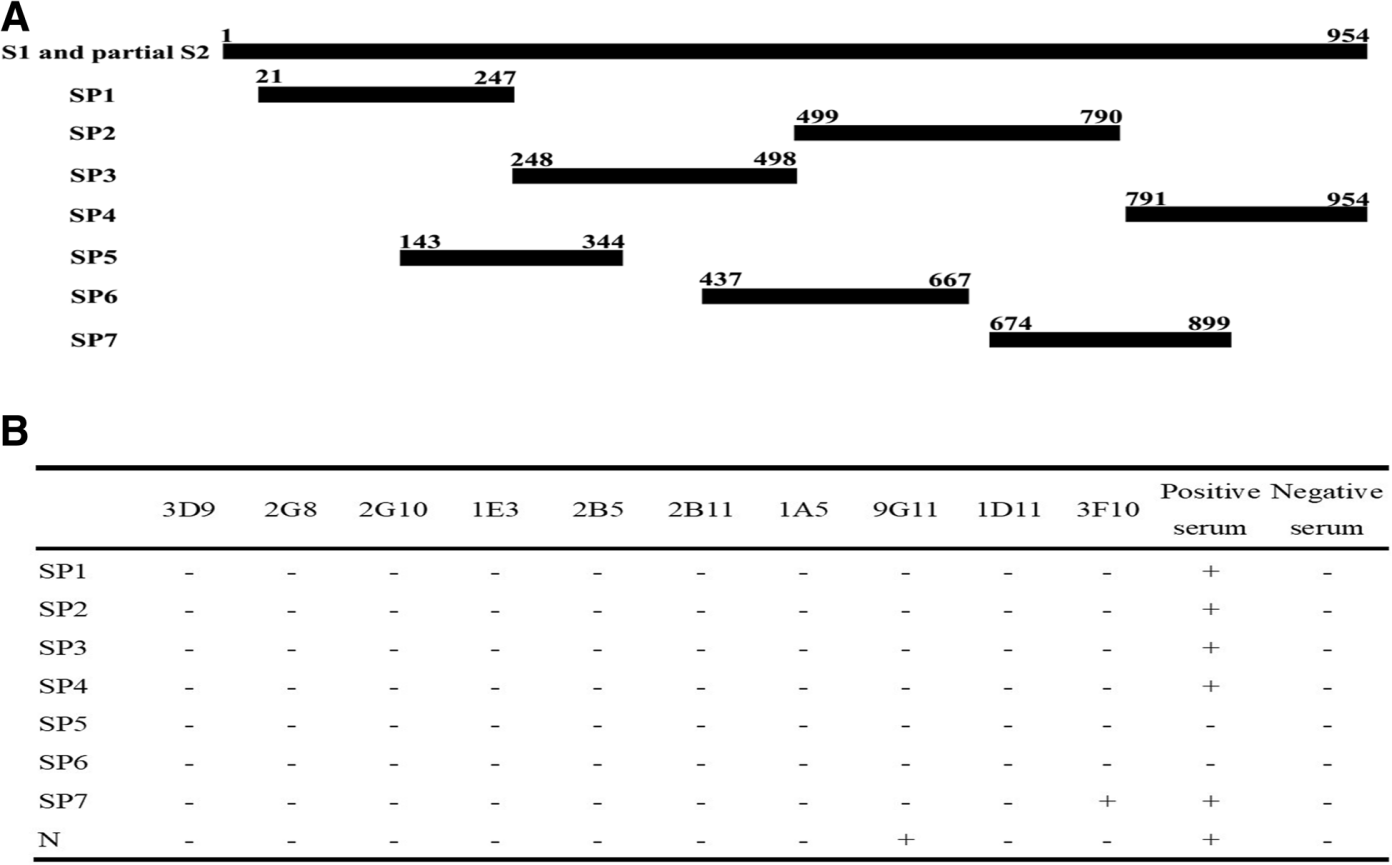
Reaction of mAb with truncated SP and N proteins tested by ELISA. **a** Schematic illustration of the location and length of the seven antigenic S fragments. **b** Summary of the mAbs binding reactivity tested by ELISA. The experiment was repeated two times

The PEDV S protein is the only identified target of PEDV nAbs. In order to further determine whether PEDV S or N protein is recognized by the mAb, the 7 truncated SP fragment (1-954aa), and full length of N were amplified, inserted into pET-32a and transformed into BL21cells. The expressed proteins were purified using a Ni-column and coated to the enzyme plate with 50 μg per well. ELISA results demonstrated that 9G11 had reactivity with N protein and 3F10 reacted with SP protein. All other mAbs didn’t react with the recombinant proteins (Fig. 2).

**Fig. 3:**
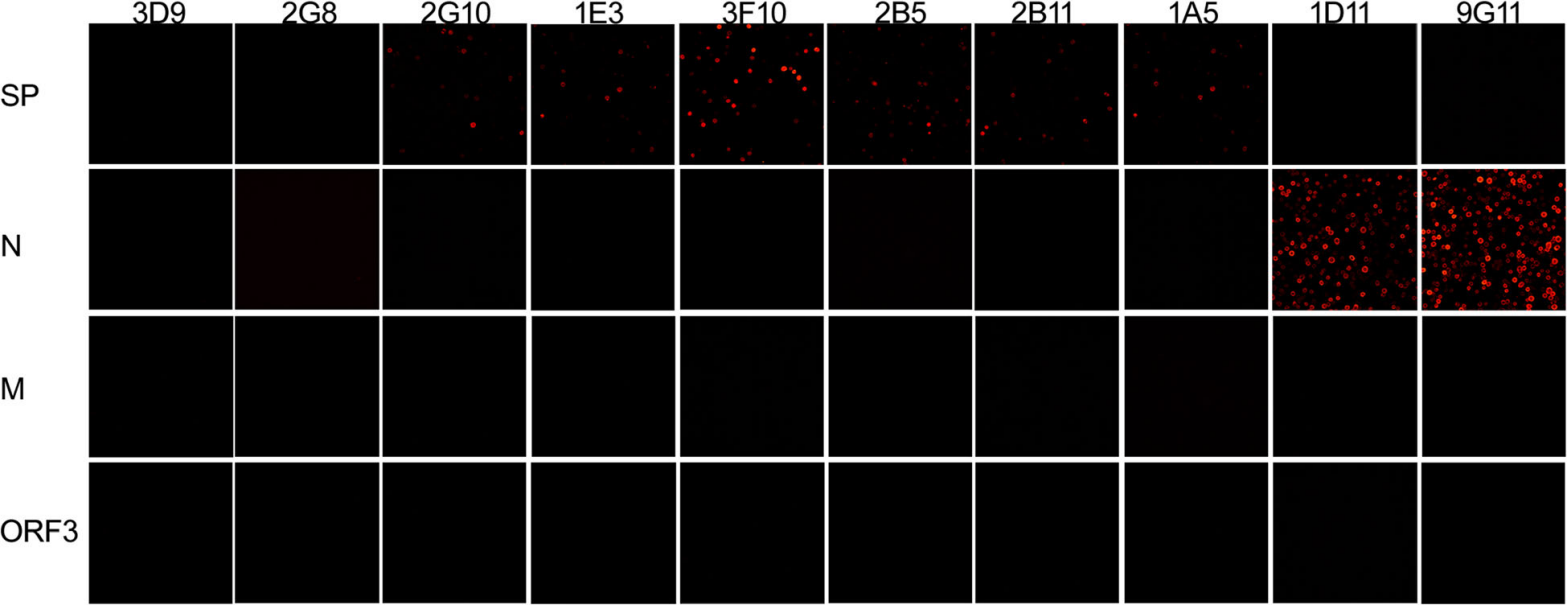
Detection of their antigenicity for mAbs by IFA. The different mAb used for the assay are indicated at the top. Recombinant protein (SP, N, M and ORF3) produced by Baculovirus expression is shown on the below. SP is the S1 and partly S2 gene fragment (encoding 1-954aa). The experiment was repeated two times, and representative images are shown

SP, N, M and ORF3 genes were also cloned into pFastBac 1 vector. Later, the sequencing results verified the expression plasmid containing SP, N, M and ORF3 in the correct direction and reading code frame, respectively. According to IFA analysis, the recombinant proteins of N, M and SP were successfully expressed in sf9 cells using mouse anti-PEDV polyclonal serum as primary antibody (see Additional file 2: Figure S2). Figure 3 shows 2B11, 1E3, 2B5, 2G10, 1A5, and 3F10 have reactivity with SP protein, 9G11 and 1D11 have reactivity with N protein, 3D9 and 2G8 have no reactivity with any protein.

### Neutralizing activity of PEDV specific mAbs

**Fig. 4:**
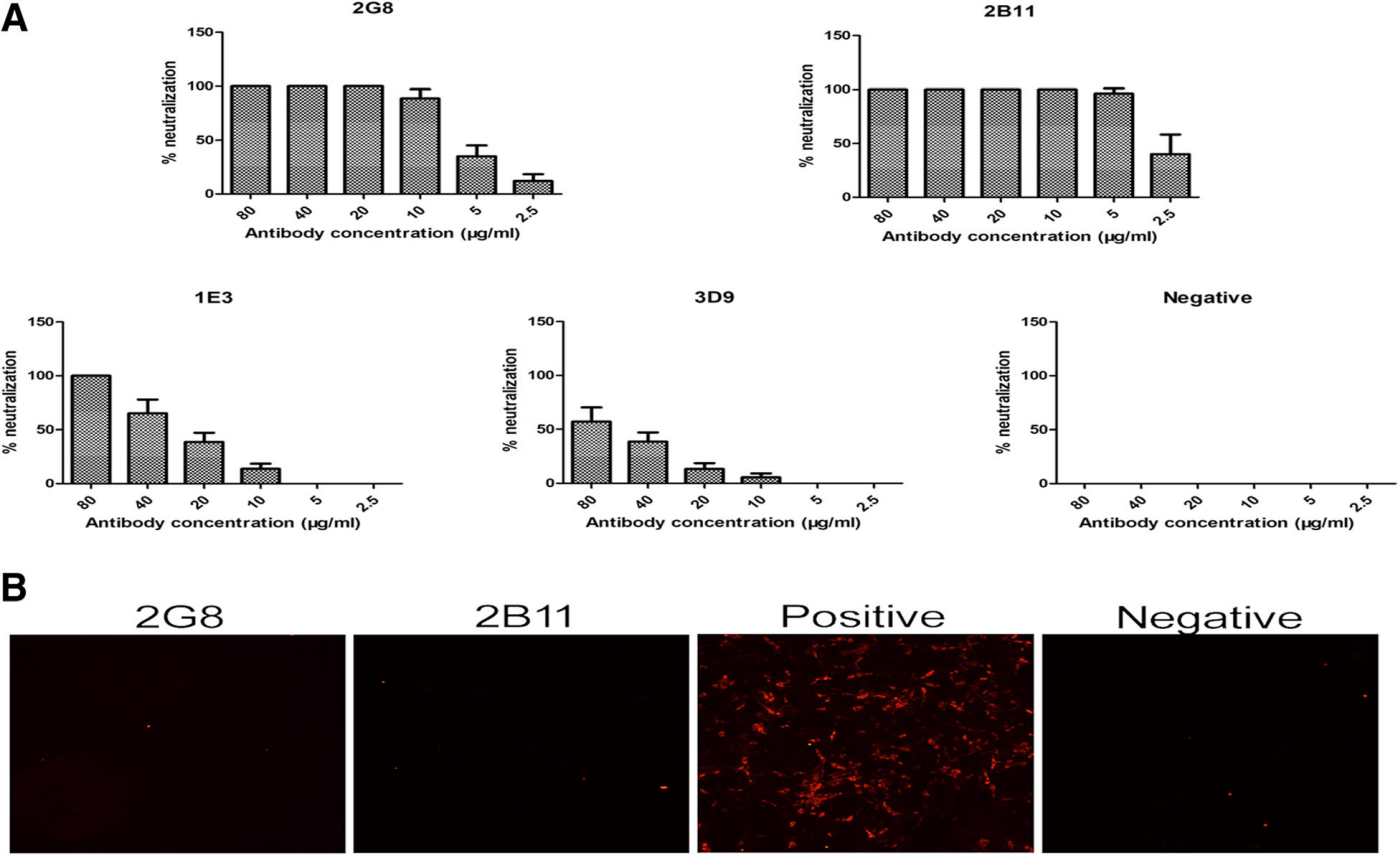
Virus neutraliztion test against PEDV-GDS01 and PEDV-GDS03. **a** Neutralization titer of antibody against GDS01. The titer of 2G8, 2B11, 1E3 and 3D9 was assessed by the plaque reduction assay. The negative serum from normal mouse was set as a negative control. **b** PEDV-GDS03-infected cells in the viral inhibition assay were detected by IFA. 9G11 targeted N protein was set a second antibody. Positive group used a negative serum and Negative group used a positive serum as control respectively. All graphs represent the means from three independent experiments. Error bars indicate standard deviations

The neutralizing activity of selected specific mAbs was assessed by PRN assays. For this, all mAbs were purified from ascites fluid by protein G and diluted to a working concentration of 80 μg/mL (in PBS). Two-fold serial dilution of the nAbs’ working stocks(80 μg/mL-2.5 μg/mL) were tested in triplicate by PRN assays. As shown in Fig. 4, 2B11, 2G8, and 1E3 completely neutralized the infection of GDS01 in Vero E6 cells at a working concentration of 10 μg/mL, 20 μg/mL and 80 μg/mL, respectively. mAb 3D9 only neutralized the 58% of the input viruses at the maximum working concentration of 80 μg/mL (Fig. 4a). All other mAbs didn’t have the ability to neutralize GDS01 virus.

In order to evaluate the neutralization effect of nAbs with the different subtype of viruses, 2G8 and 2B11 with the strongest neutralizing capacity were chosen to test the inhibition of GDS03 infection by IFA using 9G11 N protein antibody. GDS03 strain belonging to the G2 subtype doesn’t form plaque. As shown in Fig. 4b, 2G8 and 2B11 completely suppressed the GDS03 infection. These results indicated that 2G8 and 2B11 were able to inhibit the infection of both genogroups of PEDV.

### Neutralizing antibody block the attachment of PEDV to cells

**Fig. 5:**
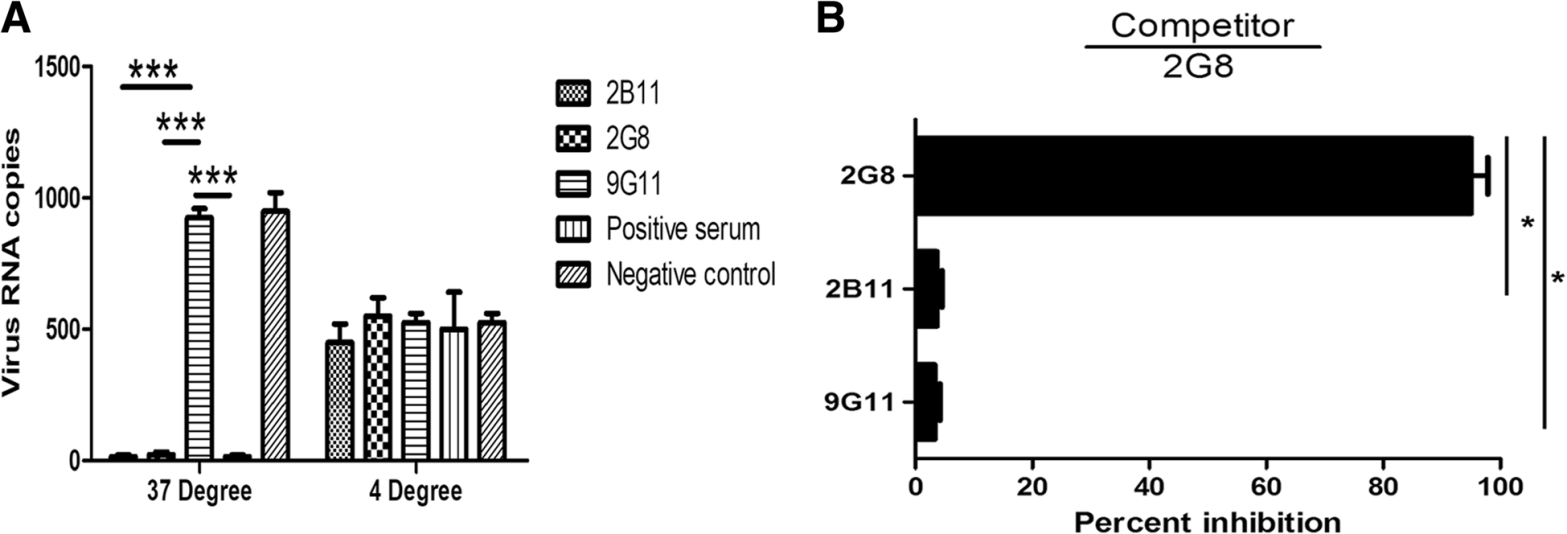
2B11 and 2G8 neutralization against PEDV interference of virus attachment to cells. **a** PEDV attachment to cells. For the 37 °C attachment, PEDV was first incubated with antibody for 1 h and then added to cells for 1 h infection incubation at 37 °C. For the 4 °C attachment, PEDV was initially incubated with Vero cells for 1 h at 4 °C, and antibody was then added to the PEDV-cell culture and the temperature was increased to 37 °C for 1 h infection incubation. The cell-associated PEDV was quantified by viral RNA RT-qPCR. 9G11 was set as an isotype control. **b** Comparison of epitopes between 2G8 and 2B11 nAbs using a competition ELISA test. 2G8 was used as a competitor, and 9G11 was used as a negative control. The results are presented as the mean ± SEM (*n* = 3). Differences were considered significant if the *P* value was < 0.05. *P* values are indicated as follows: *P < 0.05; ***P < 0.001

To explore mAbs 2B11 and 2G8 neutralizes PEDV infection in or out the cells, we tested them at the lowest concentrations. GDS01 was incubated for 1 h with antibodies at 37 °C before the mixture was added to Vero cells. At 1 h post-infection at 37 °C, the unbound viruses were washed and the cell-associated PEDVs were measured by RT-qPCR. 2B11 and 2G8 inhibited the viral entry into cells, indicating that the effect of these two nAbs was mediated by the block of viral attachment to cells (Fig. 5a). GDS01 and nAb were added to Vero cells at separate time points to further confirm the result. Firstly, PEDV was incubated with Vero cells at 4 °C for 1 h, allowing the attachment of the virus to the cell surface without entering. The cells were rinsed by PBS to remove the unattached virus. Subsequently, nAbs were added to the PEDV-cell complexes and the temperature was increased to 37 °C to initiate the viral fusion for 1 h. If the nAbs block viral attachment to cells, pre-binding of PEDV to the cells before incubation with nAb would prevent neutralization. Addition of the two nAbs after PEDV attachment to cells did not reduce cell-associated PEDV (Fig. 5a), suggesting that 2B11 and 2G8 inhibit the infection of PEDV by blocking virus attachment to cells and not downstream processes.

### Evaluation of competition of 2G8 and 2B11 binding to PEDV

Both 2G8 and 2B11 exerted their neutralization effects by directly inhibiting the tested viruses binding to the infected cells. We then determine whether the 2G8 and 2B11 antibodies bind to the same epitope. As Fig. 5b shown in competition ELISA assay, 2B11 did not compete with 2G8 for binding to PEDV. These data indicated the antibodies bind to distinct epitopes.

## Discussion

Multiple alphacoronaviruses, such as the TGEV, PRCoV, feline coronavirus type II and human coronavirus 229E(HCoV-229E), used aminopeptidase N (APN) as a receptor. But APN is not a universal receptor for the alphacoronaviruses as the human coronavirus NL63(HCoV-NL63) used angiotensin converting enzyme2(ACE2) for its entry [31, 32]. Presently, it is believed [33–35] that porcine APN acts as a functional PEDV receptor, however, whether or not pAPN is a receptor for PEDV has been debated over the years [36, 37]. Intriguingly, Vero cell lines used for isolation of PEDV strains don’t express APN that inferred from the Vero cell proteome [38]. Some data indicated that other receptors may be involved in PEDV entry into these cells, such as sialic acid and Neu5Ac [9, 39]. Isolation a nAb that inhibit virus attachment to the cell surface could help to identify the PEDV receptor.

In this study, we screened 10 mAbs through hybridoma technology. The main structural proteins of PEDV were expressed using prokaryotic and eukaryotic expression system respectively. Because immunogenic proteins were whole virus particles, the determination of the target protein of mAb is a challenge. Prokaryotic expression system expresses the products without any modification and its products are linear proteins. Baculovirus expression system has the ability to express products with glycosylation, phosphorylation and other processing modification after translation, which are similar to natural proteins. 2B11, 1E3, 2B5, 2G10 and 1A5 recognized the expressed SP protein specifically in sf9 cells but did not bind to the SP protein expressed by BL21 cells. 1D11 recognized the expressed N protein in sf9 cells but didn’t bind to the N protein expressed by BL21 cells. The results indicated that 2B11, 1E3, 2B5, 2G10, 1A5 and 1D11 specifically recognized the conformational epitope instead of the linearized epitope. 9G11 and 3F10 recognized the linearized epitope, and 2G8 and 3D9 had no reactivity with any expressed proteins. It’s possible that 2G8 and 3D9 only recognize the trimer of S protein or S2 protein. Coronavirus neutralization by antibodies is often attributed to antibody occupancy of the S trimers and interfering with viral attachment to target cells or entry. In addition, their neutralizing activity was exhibited in a dose-dependent manner. 2G8 and 2B11 have high efficiency neutralization (IC50 < 10 μg/mL), 3D9 and 1E3 have moderate neutralization (10 μg/mL < IC50 < 100 μg/mL). The observations clearly define the SP domain is most critical for PEDV to interact with its target cells.

Then anti-SP mAb 2B11 and 2G8 with the strongest neutralizing capacity were selected to explore the mechanism of nAbs. As previously reported, PEDV enters Vero cells via an initial endocytic uptake, and subsequently, the virus fuses with the PEDV S and host endosomal membrane [40]. The virus only attaches cell, but doesn’t have fusion with cell membrane at 4 °C. We found 2G8 and anti-SP mAb 2B11 efficiently bound PEDV, and then inhibited virus entry into cell at 37 °C. But if the experiment was designed into two-time points, virus infected the cells at 4 °C for 1 h, and then the mAb was added at 37 °C for 1 h, the results showed that virus could invade and replicate in cells, and the copies of virus in infected-cells had no difference regardless of the presence of 2G8, anti-SP mAb 2B11 and PEDV-negative serum. However, positive serum didn’t prevent the proliferation of intracellular viruses. This may be due to the lack of mAb in positive serum which neutralized the virus inside the cell or the interaction of mAbs makes some mAbs lose the ability of neutralization intracellular or there may exist other possible mechanisms. These results demonstrated if the viral have attached to the target cells, neutralization of 2G8 and anti-SP mAb 2B11 doesn’t work. It seems that PEDV infected cells apparently lower at 4 °C than 37 °C regardless of any antibodies, indicating that PEDV is more efficiently taken up by cells through endocytosis at 37 °C than at 4 °C. This is consistent with that the virus uptake more efficiently through endocytosis at 37 °C than at 4 °C, which was observed in Herpes simplex virus 1 infection [41]. The epitope targeted by 2G8 is completely distinct from anti-SP mAb 2B11, there may be at least two mechanisms involved neutralization effects by directly inhibiting binding to an epitope.

## Conclusions

Our study showed that 2G8 and anti-SP mAb 2B11 completely neutralize PEDV infection through blocking PEDV attachment to cells. At present, no effective prophylactic measure has been found to prevent the infection of PEDV. If the detail structure of the epitopes recognized by the two nAbs is delineated, it will be helpful for searching the new PEDV receptor and providing a new treatment method against PEDV infection.

## Additional files

Additional file 1: **Figure S1.** The integrity of virus particle checked after the ultracentrifugation. A. SDS-PAGE of PEDV, B. Western blot of PEDV. S, N and M protein were labeled by the black arrow from top to bottom respectively. M: marker, 1: sample collected from sucrose solution between 40 and 60%, 2: sample collected from sucrose solution between 20 and 40%. (TIF 6930 kb)

Additional file 2: **Figure S2.** Construction and verification of recombinant baculovirus. A. fragments of the vector and recombinant plasmid digested by EcoRI and HindIII. B. The PCR product of S, M, N and ORF3 from the recombinant baculovirus. C. Reactivity of PEDV polyclonal antibody with recombinant baculovirus infected cells, as detected by IFA. The experiment was repeated two times, and representative images are shown. (TIF 2720 kb)

## Abbreviations

DMEM: Dulbecco’s modified eagle medium
ELISA: Enzyme linked immunosorbent assay
IFA: Indirect immunofluorescence assay
mAbs: Monoclonal antibodies
nAbs: Neutralizing antibodies
PBS: Phosphate buffered saline
PEDV: Porcine epidemic diarrhea virus
