# Supplementary figures and images for: Neutralizing antibodies against porcine epidemic diarrhea virus block virus attachment and internalization
Source: Virol J. 2018 Aug 30;15:133. doi: 10.1186/s12985-018-1042-3 (PMC6117962; doi:10.1186/s12985-018-1042-3)

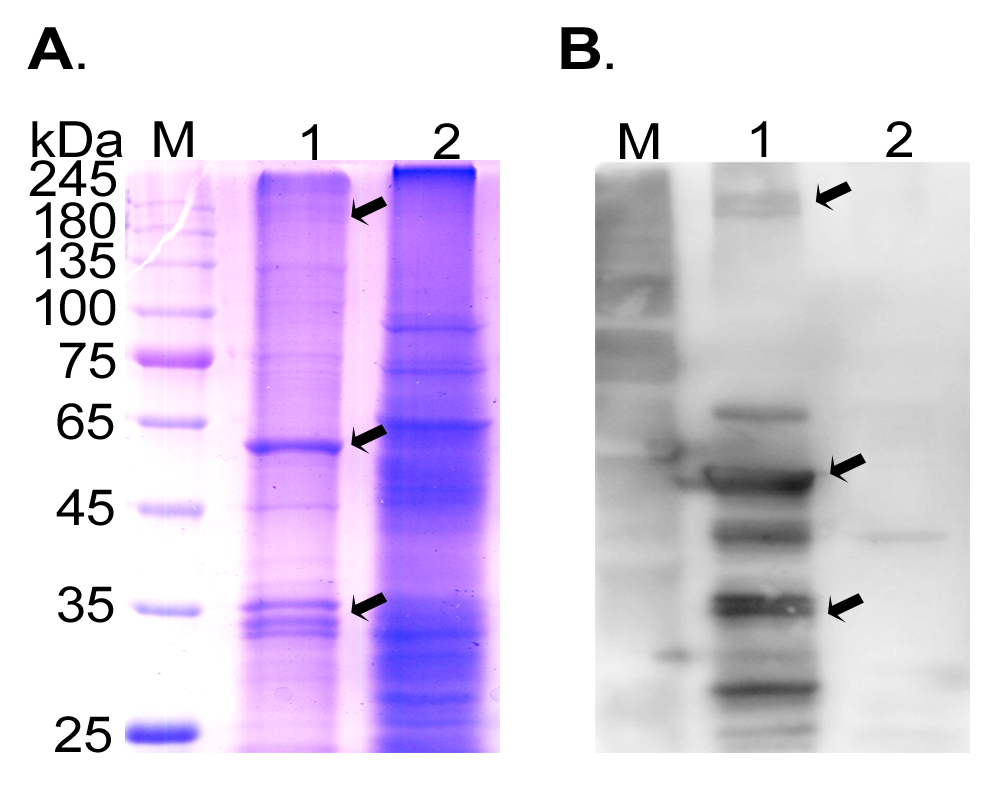

Supplement: Supplementary file 1 — Figure S1. The integrity of virus particle checked after the ultracentrifugation. A. SDS-PAGE of PEDV, B. Western blot of PEDV. S, N and M protein were labeled by the black arrow from top to bottom respectively. M: marker, 1: sample collected from sucrose solution between 40 and 60%, 2: sample collected from sucrose solution between 20 and 40%. (TIF 6930 kb) [file 12985_2018_1042_MOESM1_ESM.tif]

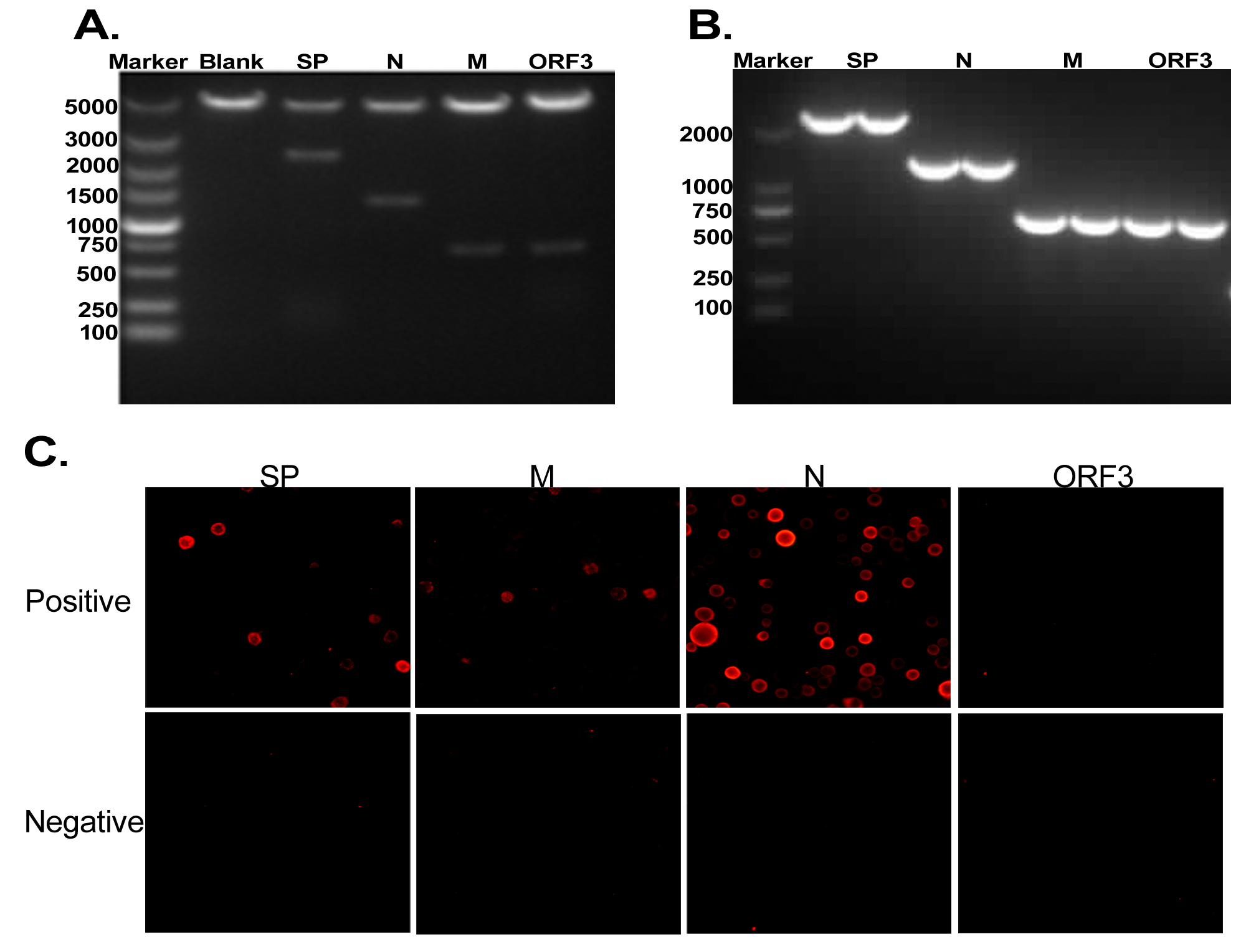

Supplement: Supplementary file 2 — Figure S2. Construction and verification of recombinant baculovirus. A. fragments of the vector and recombinant plasmid digested by EcoRI and HindIII. B. The PCR product of S, M, N and ORF3 from the recombinant baculovirus. C. Reactivity of PEDV polyclonal antibody with recombinant baculovirus infected cells, as detected by IFA. The experiment was repeated two times, and representative images are shown. (TIF 2720 kb) [file 12985_2018_1042_MOESM2_ESM.tif]
